# Quality Improvement Based on Quality Indicators Editorial on the Article “Development and Pilot-testing of Quality Indicators for Primary Care in Japan”

**DOI:** 10.31662/jmaj.2019-0018

**Published:** 2019-06-28

**Authors:** Yasuhiro Komatsu

**Affiliations:** 1 Department of Healthcare Quality and Safety, Graduate School of Medicine, Gunma University, Maebashi, Japan

**Keywords:** Quality improvement, Quality indicator, primary care

## “Knowing Is Not Enough, We Must Apply.” Johann Wolfgang von Goethe

**Figure 1.**
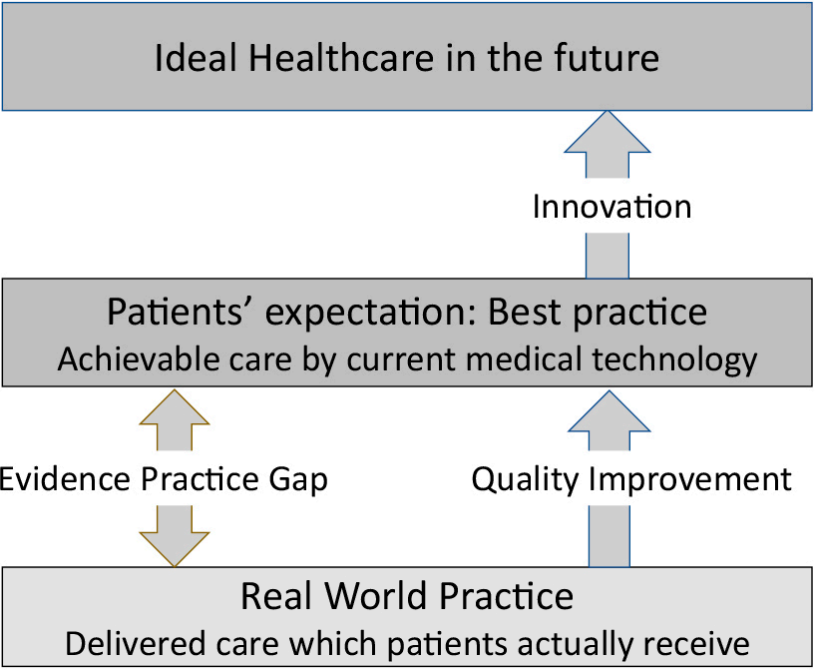
Healthcare quality and quality improvement. Healthcare quality denotes the degree to which the delivered care meets achievable best practices. As the quality increases, the gap between the best achievable care and the actual delivered care closes.

Healthcare professionals share the same desire and mission to help patients and families make changes. For certain diseases, such as advanced cancer or neurologic degenerative diseases, innovation in medical knowledge and technology are needed to improve patients’ health status further. However, for most common diseases, using standard care, if efficiently and effectively provided, greatly enhances individual and population health. We all know that smoking cessation prevents cancer and cardiovascular disease, and strict hand hygiene practice decreases healthcare-associated infections. However, we often fail to practice what we know we should do. The more advances in medical technology, the greater the gap between what the current healthcare system can achieve and what it is providing in a real world setting (Figure 1).

Hospital mission statements often include providing highest quality and safety care. There are many definitions of “quality,” but one often quoted definition is conformance to requirements, implicit or explicit needs, or expectations. Patients and the public expect to receive the best practices the current healthcare system can reasonably provide: evidence-based care which is delivered efficiently. The Institute of Medicine clearly defines healthcare quality as “the degree to which health services for individuals and populations increase the likelihood of desired health outcomes and are consistent with current professional knowledge” ^(1)^.

Integrating four phases of research can achieve best practice. 1) Basic biomedical research discovers disease pathogenesis and develops novel diagnostic and therapeutic approaches. 2) Clinical research translates basic research knowledge and provides clinical evidence. 3) Clinical epidemiology and synthesis of evidence-based medicine guide clinical practice through development of clinical practice guidelines. These guidelines are one way to close the gap between best practice and real practice; however, implementing these guidelines into everyday practice is not an easy task. There are thousands of guidelines published worldwide, and it is beyond a physician’s ability to keep up with and apply all current guidelines. 4) Quality improvement, which disseminates and applies the best available knowledge and technologies, can close the evidence practice gap to achieve quality and safety care.

Quality improvement can be defined as “the combined and unceasing efforts of everyone—healthcare professionals, patients and their families, researchers, payers, planners, and educators—to make the changes that will lead to better patient outcomes (health), better system performance (care), and better professional development (learning)” ^(2)^. The first and most critical step of quality improvement is measuring quality, through which clinicians and researchers can identify the problem and evaluate the intervention’s effectiveness. The quality indicator is a measure that assesses a particular health care process or outcome, and it can be related to the structure, process, or outcome ^(3)^. Structure indicates the attributes of the settings in which care occurs, such as facilities or presence of policy and procedure. Process indicates what is actually done, such as percentage of diabetic patients screened for microalbuminuria. The outcome indicator reflects the effect of care on patients’ health status, such as the five-year survival rate for patients receiving percutaneous coronary intervention.

Quality indicators can be used for various purposes^(3)^. Clinicians can use them for quality improvement purposes; measuring process indicators, such as the percentage of elderly patients receiving pneumococcal vaccines, can improve immunization rates among the elderly. Regulatory agencies or accreditation organizations may use quality indicators for quality assurance and nation-wide healthcare improvement. Researchers or policy makers can use them to compare the quality of care provided by primary care and hospital care providers. In Japan, patients have right to access hospitals without visiting a primary care physician, and they often seek treatment at hospitals even for minor illnesses. Robust data, based on quality indicators in primary care, can be strong evidence that care provided by primary care physicians is equivalent to, or exceeds, that provided in hospitals.

OECD published “OECD Reviews of Health Care Quality: JAPAN” in 2015 ^(4)^. The report addressed some areas for further improvement to quality of care, particularly in the areas of primary care, hospital care, and mental health care. They emphasized the importance of developing indicators linking the scope of practice defined in guidelines for primary care. In this issue of JMA Journal, Matsumura et al. reported their work on developing and pilot-testing quality indicators in Japanese primary care ^(5)^. Their excellent work corresponds to the recommendations of the OECD’s report, and will be the first step in quality improvement for primary care practice in Japan. Future practice and research on developing outcome indicators, and implementing quality improvement activities based on quality indicators will lead to the evolution of quality primary care in Japan.

## Article Information

### Conflicts of Interest

None
